# Response to Letter by J Finsterer MD

**DOI:** 10.1016/j.ebr.2024.100719

**Published:** 2024-10-24

**Authors:** Richard L. Verrier, Steven C. Schachter

**Affiliations:** Harvard Medical School, Beth Israel Deaconess Medical Center, United States; Harvard Medical School, Massachusetts General Hospital, Beth Israel Deaconess Medical Center, United States

**Keywords:** Epileptic heart syndrome, Myocardial infarction, Sudden cardiac arrest, Antiseizure medications, Cardiac stiffness, Accelerated atherosclerosis, Interictal period

To the editor:

We appreciate the thought-provoking comments by Dr. Finsterer [1] on our review of the medical literature that led us to propose the heuristic concept of the “Epileptic Heart Syndrome” [2]. While Dr. Finsterer appears to acknowledge the potential involvement of increased cardiac risk in certain patients with chronic epilepsy, he questions the rationale and utility of our syndromic approach.

The “Epileptic Heart Syndrome” classification is based on fundamental pathophysiology and a specific definition, namely, “a heart and coronary vasculature damaged by chronic epilepsy as a result of repeated surges in catecholamines and hypoxia leading to electrical and mechanical dysfunction.” It is critical to recognize that the deleterious effects of seizures are not only acute but persist during the interictal period as evidenced by measures of cardiac electrical instability such as T-wave alternans [3]. Other aspects of vascular disease also are evident in the interictal period, including carotid intima media thickening [4], stroke, transient ischemic attacks, and myocardial infarction [5], particularly in the older population.

The criteria for diagnosis of the “Epileptic Heart Syndrome” are specified in detail in Table 2 of our review [2]. These include, as foremost and essential, chronic epilepsy with or without drug resistance, as well as ECG evidence of myocardial injury, arrhythmia, and altered autonomic tone (heart rate variability measures); echocardiographic findings of diastolic dysfunction; lipid panel measures of hyperlipidemia; and accelerated atherosclerosis. These criteria are thus based on the specific pathophysiology of seizure-induced cardiac disease and are amenable to assessment with basic laboratory tests. For example, the nearly 5-fold greater incidence of myocardial infarction in patients with epilepsy compared to the general population [6] represents a major cardiac symptom that can be verified by ECG changes, echocardiography, and blood markers. Importantly, Fialho’s echocardiographic studies [7], performed in the interictal period, have revealed persistent structural changes in myocardial tissue, e.g., a chronic increase in left ventricular stiffness, consistent with the cardiotoxic effects of catecholamines released during recurring seizures.

It is essential to recognize that increased premature mortality in patients with epilepsy may not be due exclusively to SUDEP, which appears to result mainly from respiratory failure [8]. While in young individuals, <40 years of age, respiratory factors may be mainly responsible for premature death, after age 40, accelerated atherosclerosis may play a greater role, as illustrated in Figure 1 of our review [2]. As supported by Zack and Luncheon’s >95,000 respondent population survey [9], an even more extensive and independent cause of premature death in patients with epilepsy is the ∼3-fold increase in sudden arrhythmic death [10] compared to the general population.

Regarding other points raised by Dr. Finsterer, we agree that chronic epilepsy carries the potential for a neurogenic cardiomyopathy, as documented by Fialho and colleagues [11], and is more likely to be an acquired cardiomyopathy such as Takotsubo syndrome than to have a genetic basis.

Concerning the potential for adverse effects of certain antiseizure medications (ASMs), we maintain that while there can be toxic interactions with other drugs, independent effects are also significant. Specifically, ASMs with sodium-channel blocking properties, including carbamazepine, gabapentin, and phenytoin, and others, can increase risk for life-threatening arrhythmias [12]. Enzyme inducing ASMs including carbamazepine and phenytoin can contribute to hyperlipidemia and the potential for accelerated atherosclerosis, as demonstrated by Mintzer and colleagues [13]. In the 30,000-subject Canadian Longitudinal Study on Aging, Li and coworkers reported that new-onset cardiovascular events were more likely to occur in patients with epilepsy and that nearly one third of these events can be explained by the use of enzyme-inducing ASMs [5].

Based on these considerations, we believe that the cluster of pathophysiologically sound and clinically measurable parameters as specified in our syndromic approach to the “Epileptic Heart” merits further evaluation.

Sincerely,

Richard L. Verrier, Ph.D., F.H.R.S.

Steven C. Schachter, M.D.

## Ethical statement

This is a reply to a letter to the editor. It provides no new data based on clinical studies or preclinical (animal) experiments.

## CRediT authorship contribution statement

**Richard L. Verrier:** Writing – original draft, Writing – review & editing. **Steven C. Schachter:** Writing – review & editing.

## Declaration of competing interest

The authors declare the following financial interests/personal relationships which may be considered as potential competing interests: Dr. Verrier is a member of the medical advisory board of Stratus, Inc., Irving TX.

Dr. Verrier declares that he has no other known competing financial interests or personal relationships that could have appeared to influence the work reported in this paper.

Dr. Schachter declares that he has no known competing financial interests or personal relationships that could have appeared to influence the work reported in this paper.
